# Synthesis, characterization, and integrated in silico studies of 4-Aminoantipyrine-derived Schiff bases as promising antimicrobial, antioxidant, and cytotoxic agents: DFT, ADME, and molecular docking studies

**DOI:** 10.1016/j.jgeb.2026.100814

**Published:** 2026-09-10

**Authors:** Mohammed I. Sultan, Muthanna C. Urabee, Ahmed H. Sadiq, Ahmed Mutanabbi Abdula

**Affiliations:** a Quality Assurance & Performance Evaluation Department, Mustansiriyah University, Baghdad, Iraq; b Environmental Research Center, Technology University, Baghdad, Iraq; c Department of Chemistry, College of Science, Mustansiriyah University, Baghdad, Iraq

**Keywords:** Schiff base, 4-aminoantipyrine, Antibacterial, Antioxidant, Cytotoxicity, HepG2, In silico

## Abstract

This study reports the synthesis, structural characterization, experimental and computational evaluation of a series of Schiff base derivatives (**3a–3f**) obtained from 4-aminoantipyrine via condensation with various aromatic and heteroaromatic aldehydes. The synthesized compounds were fully characterized by FT-IR, ^1^H NMR, and mass spectrometry, which showed the formation of preferential imine linkages. The antibacterial activity was evaluated against *Staphylococcus aureus* and *Acinetobacter baumannii* using the agar well-diffusion method. All compounds showed significant antibacterial activity, with compound **3a** showing the highest activity against both bacterial strains (IZD = 23 mm and 20 mm, respectively). Antioxidant screening using the DPPH assay showed a wide range of radical scavenging activity, with IC_50_ values ranging from 56.3 to 423.1 μg/mL, revealing the influence of substituent electronic effects on antioxidant activity. Cytotoxicity evaluation against HepG2 cells showed that compounds 3a, 3e, and 3 f exhibited concentration-dependent cytotoxicity, with compound 3 f showing the highest inhibition (63.03% at 500 μg/mL), while exhibiting low cytotoxicity toward normal Vero cells. DFT calculations performed at the B3LYP/6-311G (d, p) level provided insight into the electronic properties and chemical reactivity of the synthesized compounds, while the Absorption, Distribution, Metabolism, and Excretion (ADME) predictions suggested favorable pharmacokinetic profiles. Molecular docking studies showed strong binding affinities for DNA gyrase (PDB ID: 4DUH) and VEGFR2 kinase (PDB ID: 3VHE), with compound **3e** showing the highest binding affinity for DNA gyrase (−8.2 kcal/mol) and compound **3a**, **3e**, and **3** **f** exhibited the highest binding affinities toward VEGFR2 (−10.3 kcal/mol). The determined biological activities were consistent with the docking results, supporting these derivatives as promising multifunctional scaffolds for the development of novel antibacterial and anticancer agents.

## 1 Introduction

Bacterial infections, accompanied by a rapid increase in antimicrobial resistance (AMR),^1^ significantly reduce the efficacy of conventional antibiotics and contribute to increased morbidity and mortality.^2^ This highlights the urgent need for the development of new therapeutic agents with improved selectivity, efficacy, and pharmacokinetic profiles.^3^ Oxidative stress resulting from an imbalance between reactive oxygen species (ROS) and antioxidant defense mechanisms plays an important role in the pathogenesis of various diseases, including cancer and infectious diseases.^4^ Excessive production of reactive species can result in cell death, DNA mutations, and abnormal cell proliferation.^5^ Therefore, antioxidant compounds are of great interest as they can be combined with antimicrobial and anticancer agents to reduce oxidative stress and may enhance therapeutic outcomes^6^.^7^ Meanwhile, cancer remains one of the leading causes of mortality worldwide, causing hundreds of thousands of deaths each year and placing a significant burden on healthcare systems, especially in developing countries.^8^ Despite major advances in therapeutic technologies, including chemotherapy,^9^ radiotherapy,^10^ and targeted therapy,^11^ the efficacy of current therapies is often limited by severe side effects, exorbitant costs, and the emergence of drug resistance.^12^ Recent advances in synthetic biology, genome mining, and metagenomics have provided promising strategies for the sustainable discovery and production of bioactive molecules.^13^ Schiff bases have attracted great interest due to their structural diversity and wide spectrum of biological activities. These compounds are characterized by the presence of azomethine linkage (–C=N–) and are noted for ease of synthesis and structural robustness. Notably, Schiff bases derived from 4-aminoantipyrine (4-AAP) have established promising pharmaceutical activity, including antiviral,^14^ antifungal,^15^ antibacterial,^16^ antioxidant,^17^ anti-inflammatory,^18^ and anticancer.^16^ Furthermore, the presence of pyrazolone moiety in 4-aminoantipyrine enhances the biological potential of their derivatives by facilitating the binding interactions with biological targets.^19^ Recent advances in computational chemistry have significantly improved the discovery and optimization of bioactive molecules. Techniques including density functional theory (DFT), molecular docking, and ADME prediction are becoming increasingly important tools for structure–activity relationships,^20^ pharmacokinetic behavior, and ligand-target interactions at the molecular level. In particular, the molecular docking studies focused on key enzymes, including DNA gyrase, an important bacterial enzyme involved in DNA replication,^21^ and vascular endothelial growth factor receptor-2 (VEGFR2), an important regulator of angiogenesis and tumor progression,^22^ provide valuable insights into the potential mechanisms of action of newly synthesized compounds.

The current work is based on the structural modification of 4-aminoantipyrine through Schiff base formation with different aromatic and heteroaromatic aldehydes that can produce new skeletons with potentially enhanced antibacterial, antioxidant, and cytotoxic activities. Furthermore, the variations in the electronic and physicochemical properties of modified Schiff bases influence their biological activity, molecular recognition, and binding affinity toward DNA gyrase and VEGFR2, thereby providing valuable structure–property–activity relationship (SPAR) insights for the design of new bioactive molecules. The present study provides a comprehensive experimental and computational evaluation of these derivatives, establishing correlations between their structural features, physicochemical properties, and biological activities, thereby contributing to the rational development of bioactive Schiff base derivatives.

**Fig. 1:**
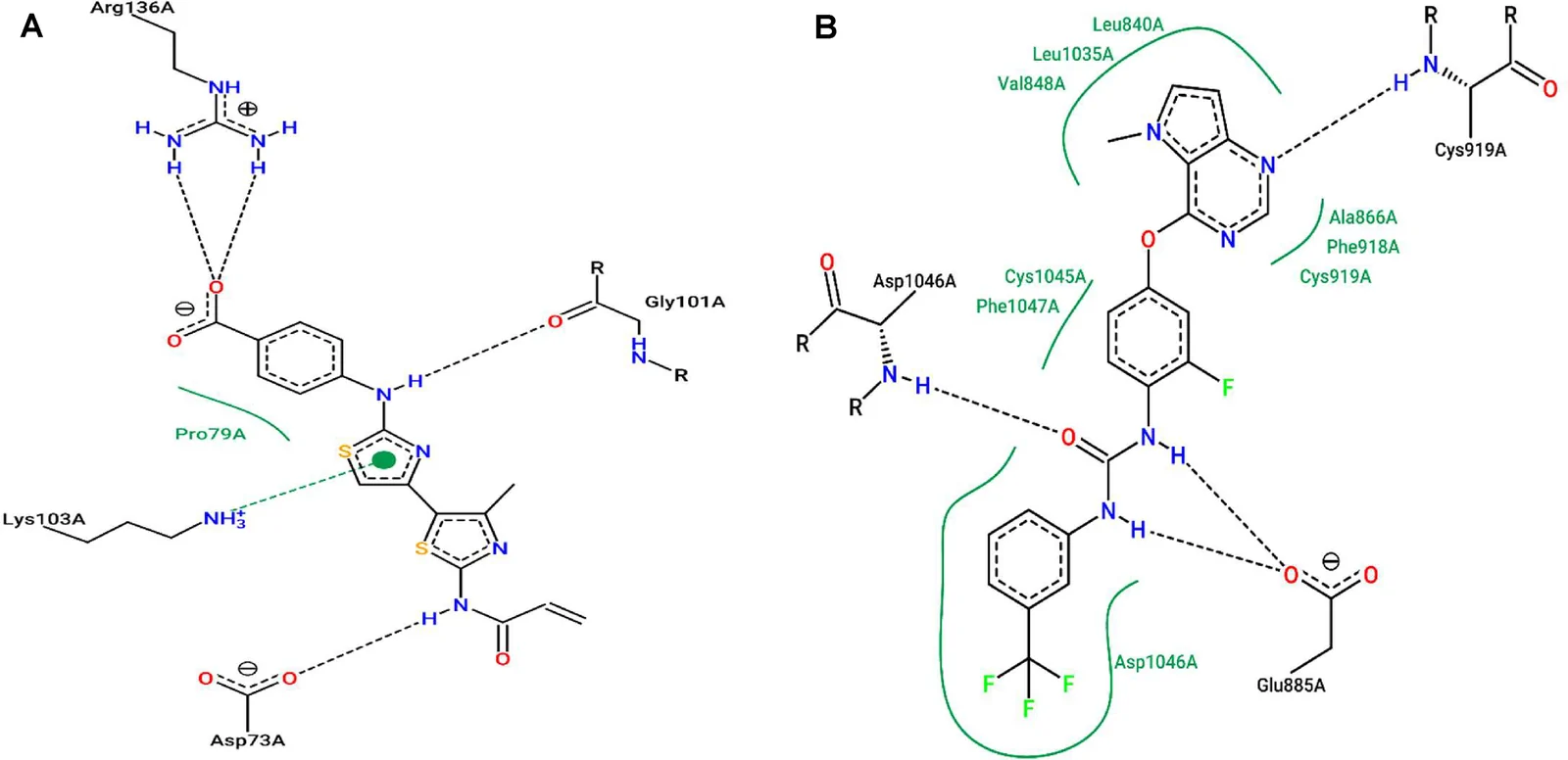
The active sites of the proteins under study were marked in 2D diagrams: A. DNA gyrase's active site displaying interactions with the co-crystallized ligand (4-{[4′-methyl-2′-(propanoylamino)-4,5′-bi-1,3-thiazol-2-yl]amino}benzoic acid); B. VEGFR2 kinase's active site demonstrating the interactions between the co-crystallized ligand (1-{2-fluoro-4-[(5-methyl-5H-pyrrolo[3,2-*d*]pyrimidin-4-yl)oxy]phenyl}-3-[3-(trifluoromethyl)phenyl]urea) (Obtained from Proteins Plus, a pose view website, at https://proteins.plus/ (accessed on March 25, 2026).

Therefore, a series of Schiff base derivatives (**3a–3f**) based on 4-aminoantipyrine were synthesized in good yields and pharmacologically evaluated for their antibacterial activity against both *Staphylococcus aureus* (Gram-positive) and *Acinetobacter baumannii* (Gram-negative), as well as for their antioxidant capacity using the DPPH radical scavenging assay. Furthermore, the selected derivatives were evaluated for their cytotoxic activity against HepG2 cancer cell line and normal Vero cells to determine their selectivity profile. In silico studies, including DFT calculations, molecular electrostatic potential (MEP) analysis, ADME prediction, and molecular docking, were performed. Docking studies against DNA gyrase involved key binding site residues, namely Arg136, Lys103, Gly101, Pro79, and Asp73,^23^ while VEGFR2 interactions involved Phe1047, Asp1046, Cys1045, Leu1035, Cys919, Phe918, Glu885, Ala866, Val848, and Leu840,^24^ as illustrated in Fig. 1A and B, respectively.

## 2 Results and discussion

### 2.1 Chemistry

**Scheme 1:**
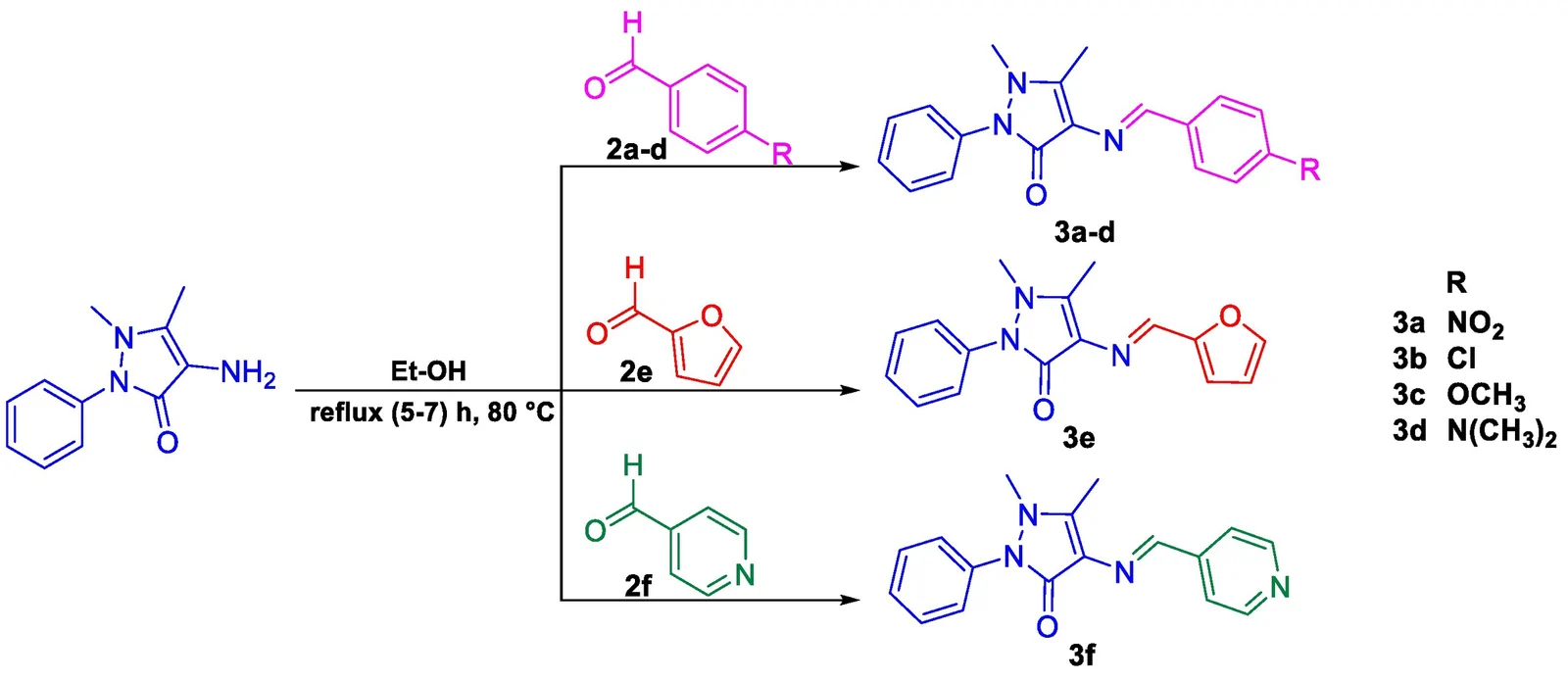
Synthesis of Schiff Bases compounds (**3a–3f**) derived from 4-aminoantipyrine.

Schiff bases (**3a–3f**) were synthesized by reacting 4-aminoantipyrine (4-AAP) with various para-substituted benzaldehydes (**2a–2d**), furan-2-carbaldehyde (**2e**), and pyridine-4-carbaldehyde (**2f**) using the previously published protocol^16^ as shown in Scheme 1. The structures of the synthesized Schiff base derivatives (**3a–3f**) were characterized using different techniques including FT-IR, ^1^H NMR, and mass spectrometry.

For compound **3a**, the FT-IR spectrum showed characteristic bands at 1638 cm^−1^ corresponding to the νC=O group of the antipyrine ring and at 1596 cm^−1^ attributed to the azomethine (C=N) stretching vibration, confirming Schiff base formation. Additional bands at 1550 and 1335 cm^−1^ were assigned to the asymmetric and symmetric stretching vibrations of the NO₂ group, respectively. In the ^1^H NMR spectrum, a singlet at *δ* 9.66 ppm was assigned to the -N=CH proton, while two doublets at *δ* 8.27 and 8.04 ppm (*J* = 9 Hz) corresponded to the para-substituted aromatic ring. The N–CH_3_ and C–CH_3_ protons appeared at 3.30 and 3.25 ppm, respectively. The molecular ion peak at *m*/*z* 336.3 confirmed the molecular formula C_18_H_16_N_4_O_3_.

The FT-IR spectrum of compound **3e** showed characteristic stretching bands at 1642 cm^−1^ ν(C=O) and 1590 cm^−1^ ν(C=N), along with aromatic stretching ν(C—H) absorption in the 3110–3045 cm^−1^ range. The ^1^H NMR spectrum showed a singlet at *δ* 9.44 ppm corresponding to the azomethine proton. Furthermore, the furan ring protons appeared at *δ* 7.84, 6.95 and 6.63 ppm, and the phenyl protons were observed between *δ* 7.53–7.35 ppm, and N–CH₃ and C–CH₃ signals appeared at *δ* 3.16 and 2.40 ppm, respectively. The molecular ion peak at *m*/*z* 281.2 is consistent with the formula C₁_6_H₁₅N₃O₂. On the other hand, FT-IR spectrum of compound **3** **f** showed absorption bands at 1639 cm^−1^ for the C=O group and 1595 cm^−1^ for C=N stretching vibrations. In the ^1^H NMR spectrum, the azomethine proton appeared as a singlet at *δ* 9.56 ppm, while the pyridine ring protons were observed as two doublets at *δ* 8.65 and 7.74 ppm (*J* = 6.3 Hz). Phenyl protons appeared between *δ* 7.56–7.38 ppm, and N–CH₃ and C–CH₃ groups were found at *δ* 3.32 and 3.25 ppm, respectively. The molecular ion peak at *m*/*z* 292.2 confirmed the C_17_H_16_N_4_O molecular formula.

Overall, spectroscopic data obtained from FT-IR, ^1^H NMR and mass spectrometry were consistent with the proposed structures of the synthesized Schiff base derivatives.

### 2.2 Biology

#### 2.2.1 Antibacterial activity of the Schiff bases derived from 4-aminoantipyrine (**3a–3f**)

**Fig. 2:**
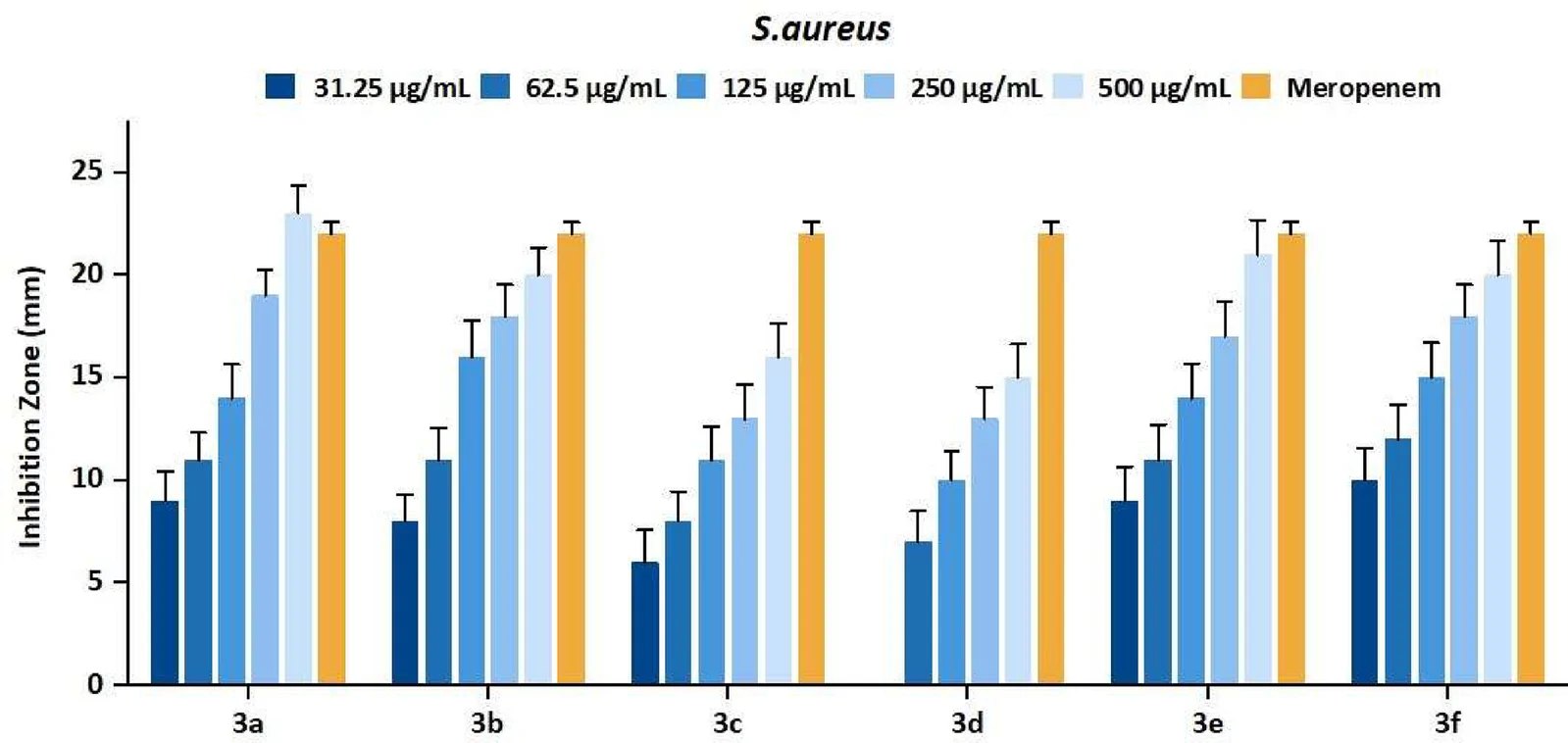
Inhibition zones of compounds **3a–3f** against the Gram-positive *S.aureus*. Data are presented as mean ± SD of three independent experiments (*n* = 3).

**Fig. 3:**
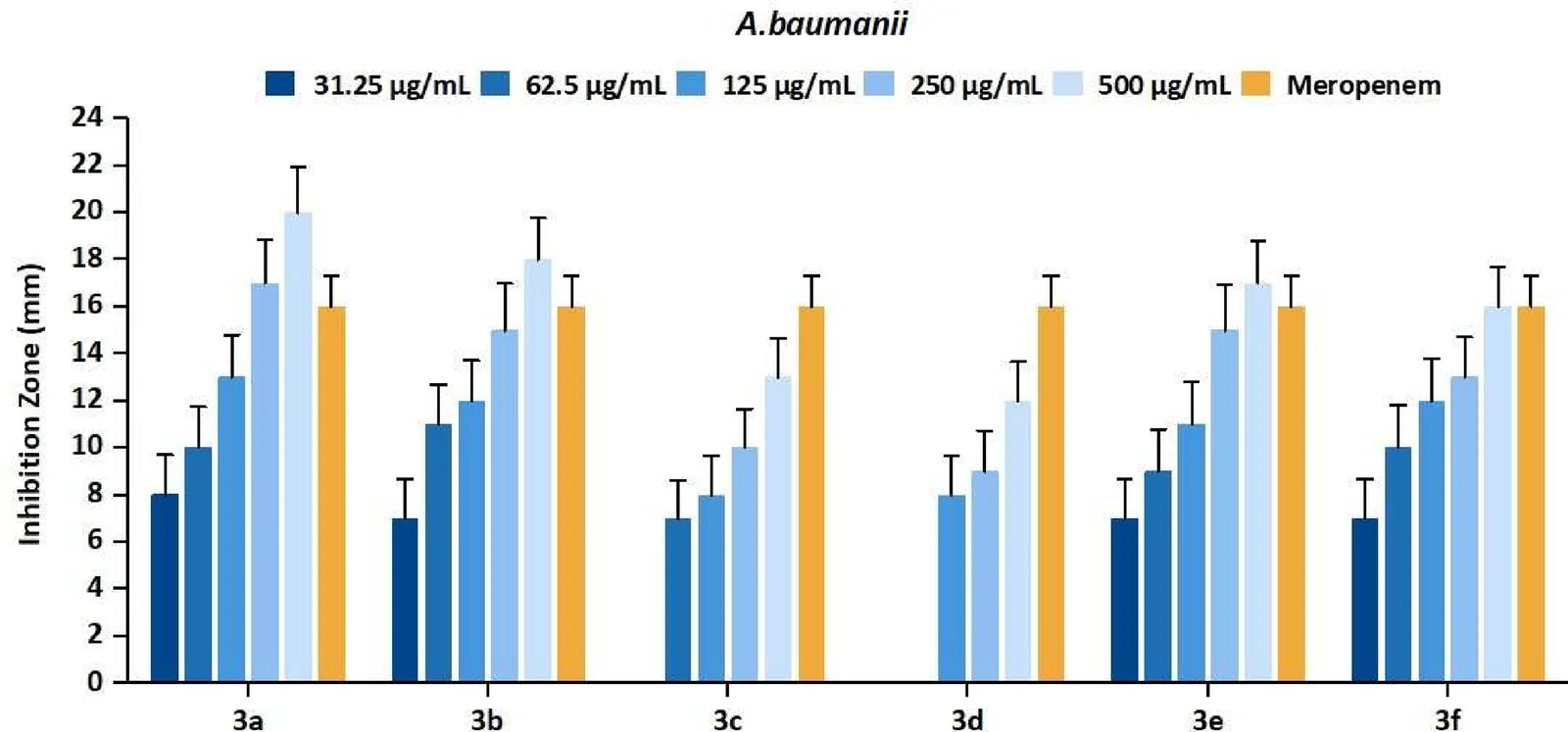
Inhibition zones of compounds **3a–3f** against Gram-negative *A. baumannii*. Data are presented as mean ± SD of three independent experiments (*n* = 3).

The inhibition zone diameters (IZD) of the synthesized compounds (**3a–3f)** against *S. aureus* (Gram-positive) and *A. baumannii* (Gram-negative), determined using the agar well-diffusion method at 31.25–500 μg/mL, are presented in Fig. 2, Fig. 3. All compounds displayed concentration-dependent antibacterial activity against both strains, with meropenem (IZD = 22 mm for *S. aureus*; 16 mm for *A. baumannii*) and DMSO serving as positive and negative controls, respectively. Against *S. aureus*, compound **3a** exhibited the highest inhibition zone among the synthesized compounds (23 mm at 500 μg/mL), followed by **3e** (21 mm), **3b** (20 mm), **3** **f** (20 mm), **3c** (16 mm), and **3d** (15 mm). Overall inhibition zones against *A. baumannii* were lower than those against *S. aureus* across the entire series, with the overall antibacterial activity order being: **3a > 3b > 3e ≈ 3** **f > 3c > 3d**.

The antibacterial activity of the synthesized Schiff base derivatives (**3a-f**) against *A. baumannii* and *S. aureus* can be rationalized through the biological activity of the C=N linkage, which exerts antibacterial effects via disruption of bacterial membrane integrity and potential inhibition of DNA gyrase.^26^ The experimental results revealed a clear structure–activity relationship (SAR), with electron-withdrawing substituents being associated with higher antibacterial activity in this series. Compound **3a** was the most potent derivative against both strains, a result attributable to the electron-withdrawing effect of the –NO_2_ group, which enhances electrophilicity of the azomethine system and may enhance interactions with biological targets,^25^.^26^ Compound **3b** ranked second, consistent with the role of halogen substituents in membrane permeation through increased lipophilicity.^29^ Compounds **3e** and **3** **f** exhibited moderate activity which may be attributed to their ability to form hydrogen bonds through their heteroaromatic rings with bacterial enzyme-active sites. In contrast, compounds 3c (–OCH₃) and 3d [–N(CH₃)₂], bearing electron-donating substituents, exhibited the weakest antibacterial activity against both strains, because those substituents may reduce the normal lipophilicity and prevent diffusion through all bacterial membranes,^27^.^28^ The lower inhibition activity observed against *A. baumannii* compared to *S. aureus* can be related to the additional permeability barrier implied by the use of the outer membrane by Gram-negative bacteria,^29^.^30^ The antibacterial activity of the synthesized derivatives is consistent with recent publications describing 4-aminoantipyrine-based Schiff bases as promising antimicrobial agents. Previous studies have illustrated that the incorporation of aromatic and heteroaromatic moieties into the 4-aminoantipyrine skeleton enhances antibacterial activity by improving molecular interactions with bacterial targets, particularly DNA gyrase. The antibacterial activity of Schiff bases in the present study is therefore in good agreement with these findings, suggesting that the electronic nature and structural features of the aromatic and heterocyclic substituents play important roles in determining antibacterial potency.^31^

#### 2.2.2 DPPH assay: Antioxidant activity of Schiff base derived from 4-aminoantipyrine (**3a–3f**)

**Fig. 4:**
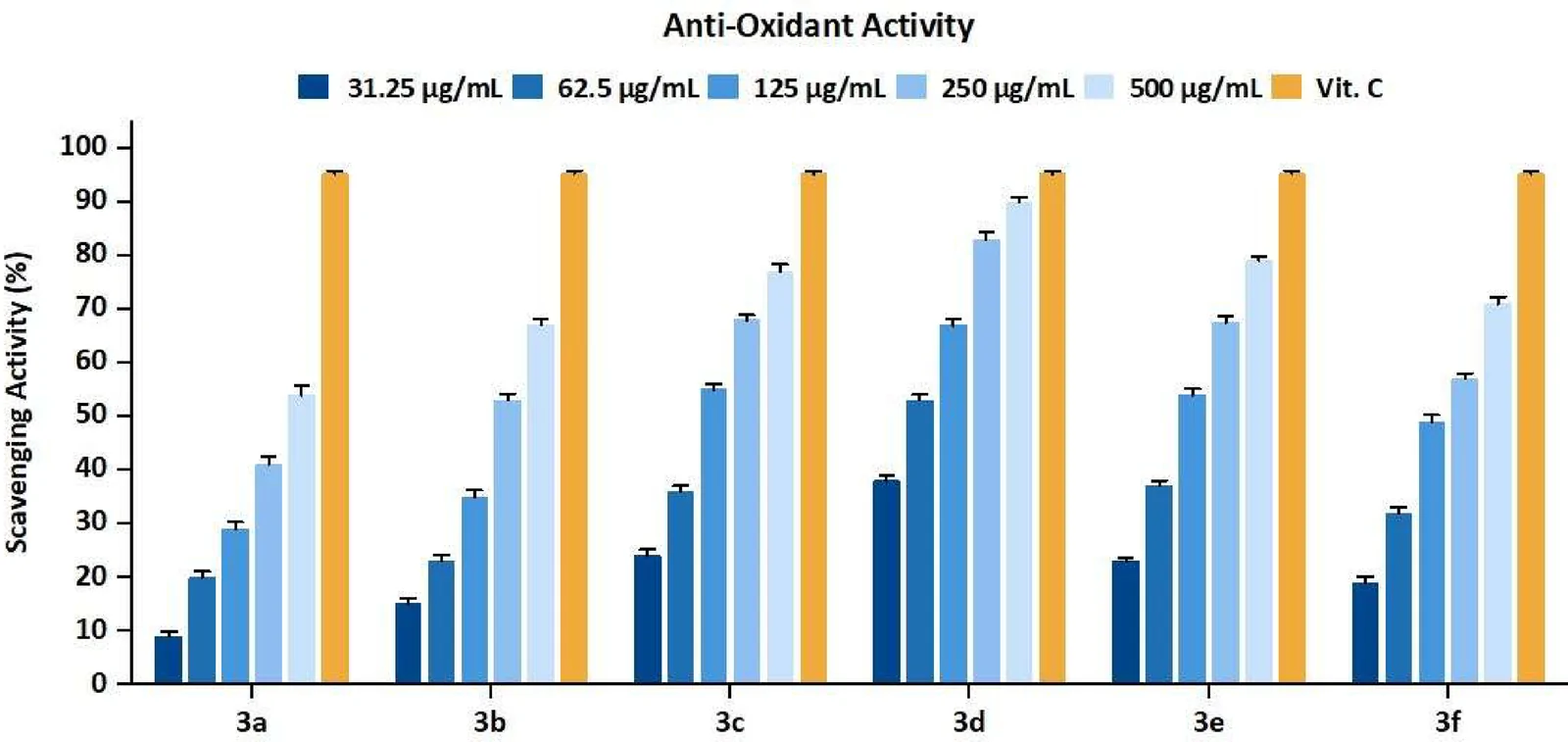
Free radical scavenging activity of compounds **3a–3f**, at different concentrations (31.25–500 μg/mL). Data are presented as mean ± SD of three independent experiments (*n* = 3).

The antioxidant capacity of newly synthesized Schiff-base derivatives (**3a–3f**) was evaluated using the 2,2-diphenyl-1-picrylhydrazyl (DPPH) radical assay in vitro at five concentrations (31.25, 62.5, 125, 250, and 500 μg/mL) in DMSO. Free radical scavenging assay results are summarized in Fig. 4. All derivatives showed concentration-dependent radical scavenging activity, as evidenced by the gradual increase in scavenging activity with increasing concentration. The half-maximal inhibitory concentration (IC_50_) values ranged from 56.3 to 423.1 μg/mL, which reflects marked differences in activity as a function of the substitution pattern of the aromatic ring.

Generally, all compounds exhibited concentration-dependent radical scavenging activity. The highest efficiency was shown by compound **3d** (IC_50_ = 56.3 μg/mL, 90% inhibition at 500 μg/mL) due to the strong electron-donating effect of the 4-(dimethylamino) group, which increases electron density through resonance and facilitates atom transfer to DPPH radical,^32^.^33^ Compound **3c** showed an IC_50_ value of 108.6 μg/mL, which is consistent with the ability of methoxy groups to donate hydrogen atoms via HAT and SPLET mechanisms,^34^.^35^ This suggests the electronic order: -N(CH_3_)_2_ **>** -OCH_3_. Compound **3e** (furan-2-yl, IC_50_ = 110.3 μg/mL) and **3** **f** (pyridin-4-yl, IC_50_ = 140.6 μg/mL) showed moderate activity; The furan oxygen contributes to lone-pair delocalization while the electron-withdrawing pyridine nitrogen reduces the available electron density, thereby reducing antiradical efficiency.^36^ Compound **3b** (—Cl, IC_50_ = 229.2 μg/mL) showed an intermediate value, while Compound **3a**, bearing a strong electron-withdrawing nitro group showed the lowest activity (-NO_2_, IC_50_ = 423.1 μg/mL). In pyrazolone Schiff base series, nitro substitution consistently leads to poor antiradical performance,^37^.^38^ The SAR data are consistent with recent research indicating that electron-donating para-substituents may contribute to enhanced DPPH radical-scavenging activity in 4-aminopyrazolone Schiff base derivatives,^32^.^38^ The antioxidant outcomes from the current study are comparable with recent results on Schiff base derivatives, which illustrated that electron-donating and conjugated substituents contribute to enhanced free radical scavenging activity. The concentration-dependent DPPH scavenging observed for the synthesized Schiff bases supports the proposed relationship between molecular structure and antioxidant activity and is consistent with recent reports describing Schiff bases as effective synthetic antioxidants.^39^

#### 2.2.3 MTT assay: Cytotoxicity of the Schiff base derivatives (**3a**, **3e**, and **3** **f**) against HepG2 cells

**Fig. 5:**
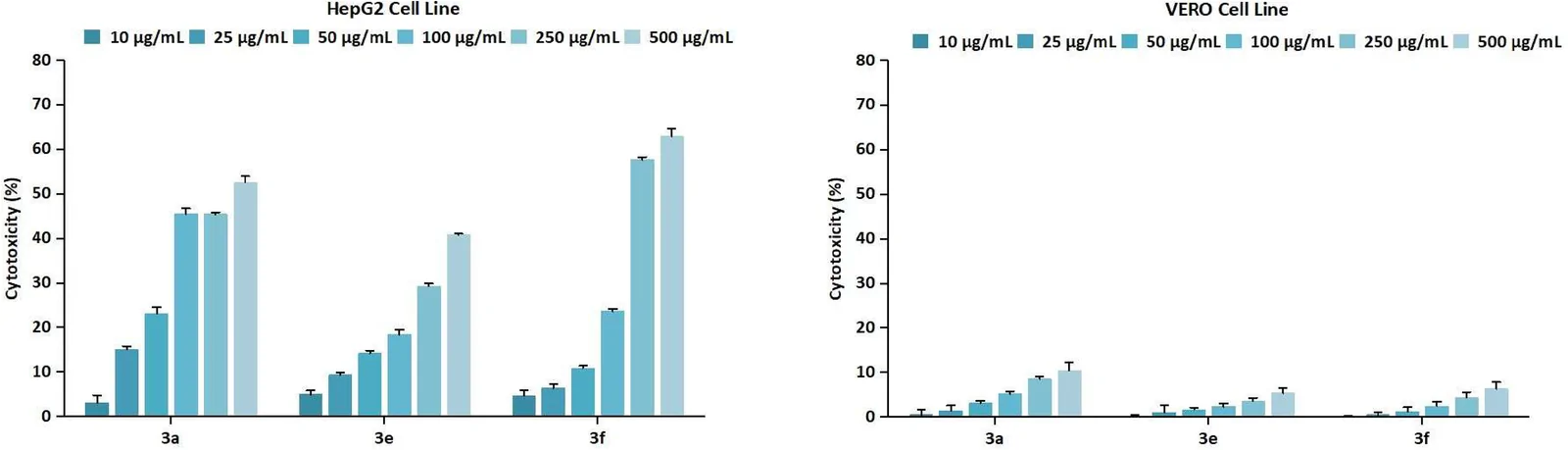
Cytotoxic activity of 4-aminoantipyrine derivatives (**3a**, **3e**, and **3** **f**). A. HepG2 cell line and B. VERO cell line. Data are presented as mean ± SD of three independent experiments (*n* = 3). Statistical analysis of the cytotoxicity data was performed using one-way ANOVA followed by Tukey's multiple comparison test, which demonstrated significant differences among treatment groups (***p* < 0.0001**).

**Fig. 6:**
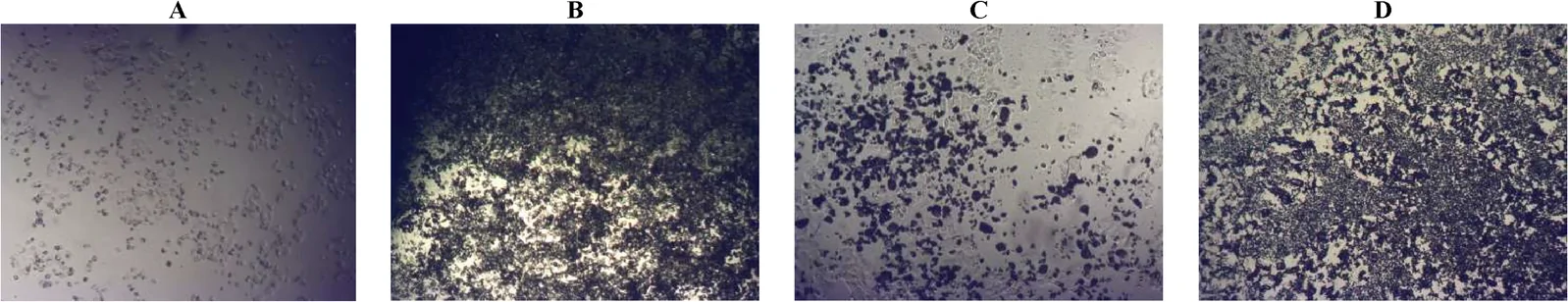
HepG2 cell line apoptosis indicators after 4-aminoantipyrine derivatives treatment (**3a**, **3e**, and **3** **f**). **A.** Control cells; **B.** cells treated with **3a**; **C.** cells treated with **3e**; and **D.** cells treated with **3** **f**, scale bar: 100 μm; test substance concentration: 500 μg/mL.

Cytotoxic activity of compounds **3a**, **3e**, and **3** **f** was evaluated against human hepatocellular carcinoma cell line HepG2 and non-carcinogenic Vero cell line using MTT assay at concentrations of 10–500 μg/mL over 48 h as shown in Fig. 5. All three compounds demonstrated a clear concentration-dependent decrease in HepG2 cell viability (Fig. 5A), indicating concentration-dependent cytotoxic effects.^40^,^41^.^42^ Inhibition values were modest at 10 μg/mL (3.2–5.1%) and increased progressively, reaching 40.85–63.03% at 500 μg/mL, reflecting the dose-response profile characteristic of compounds acting through concentration-dependent disruption of mitochondrial metabolic activity.^43^ Representative morphological changes in HepG2 cells following treatment with compounds 3a, 3e, and 3f at 500 μg/mL are shown in Fig. 6

Among the evaluated derivatives, compound **3** **f** showed the highest cytotoxic potency (63.03% inhibition at 500 μg/mL) with an IC_50_ = 291.6 μg/mL. The relatively high cytotoxicity of **3** **f** may be associated with structural features of the pyridine-containing Schiff base; however, the underlying mechanism cannot be established from the present cell-viability assay^44^.^45^ It is consistent with reports confirming that pyridine-containing Schiff bases consistently show better anti-proliferative activity against HepG2, MCF-7, and HeLa cell lines compared to their benzylidene counterparts.^45^ Compound **3a** ranked second in cytotoxic activity, exhibiting 53.77% inhibition at 500 μg/mL with an IC₅₀ value of 429.0 μg/mL. The strongly electron-withdrawing nitro group enhances the electrophilic character of the azomethine device, promotes interactions with electron-rich cellular nucleophiles including DNA bases and thiol-containing enzymes, and may facilitate interactions with DNA^46^. Compound **3e** showed the lowest activity (40.85% inhibition at 500 μg/mL), presumably due to the higher electron density of the furan ring, which can also modulate membrane permeability and reduce intracellular target affinity. Nevertheless, its smooth dose-response behavior is consistent with reports describing furan-containing heterocyclic compounds with cytotoxic or antiproliferative activity.^47^

An important finding is the pronounced selectivity toward HepG2 cells over the normal Vero cell line, where maximum inhibition values were only 10.48%, 5.49%, and 6.43% for compounds **3a**, **3e**, and **3** **f**, respectively, at 500 μg/mL (Fig. 5B). This differential cytotoxic response suggests a potentially favorable selectivity profile and warrants further investigation using additional cancer and normal cell models^40^.^48^ In comparison, the present free ligands showed moderate activity relative to reported Mn(II) Schiff base complexes, which achieved IC50 values as low as 2.6 μg/mL against HepG2 cells.^49^ Metal complexation has been reported to enhance lipophilicity, cellular uptake, and DNA-binding capacity^42^.

The overall SAR order (compound **3** **f > 3a > 3e**) reflects the combined influence of electronic character and π-conjugation extent on the antiproliferative potency of the pyrazolone–azomethine pharmacophore and provides a rationale for further developing the corresponding metal complexes and mechanistic studies of apoptosis and molecular docking studies. The selective cytotoxic activity of synthesized compounds toward HepG2 cells is in agreement with recent studies demonstrating that 4-aminoantipyrine-derived Schiff bases exhibit promising antiproliferative activity against human cancer cell lines while maintaining relatively low toxicity toward normal cells.^50^

### 2.3 In silico study

#### 2.3.1 Global reactivity descriptors and molecular electrostatic potential (MEP) of the synthesized compounds (**3a–3f**)

**Fig. 7:**
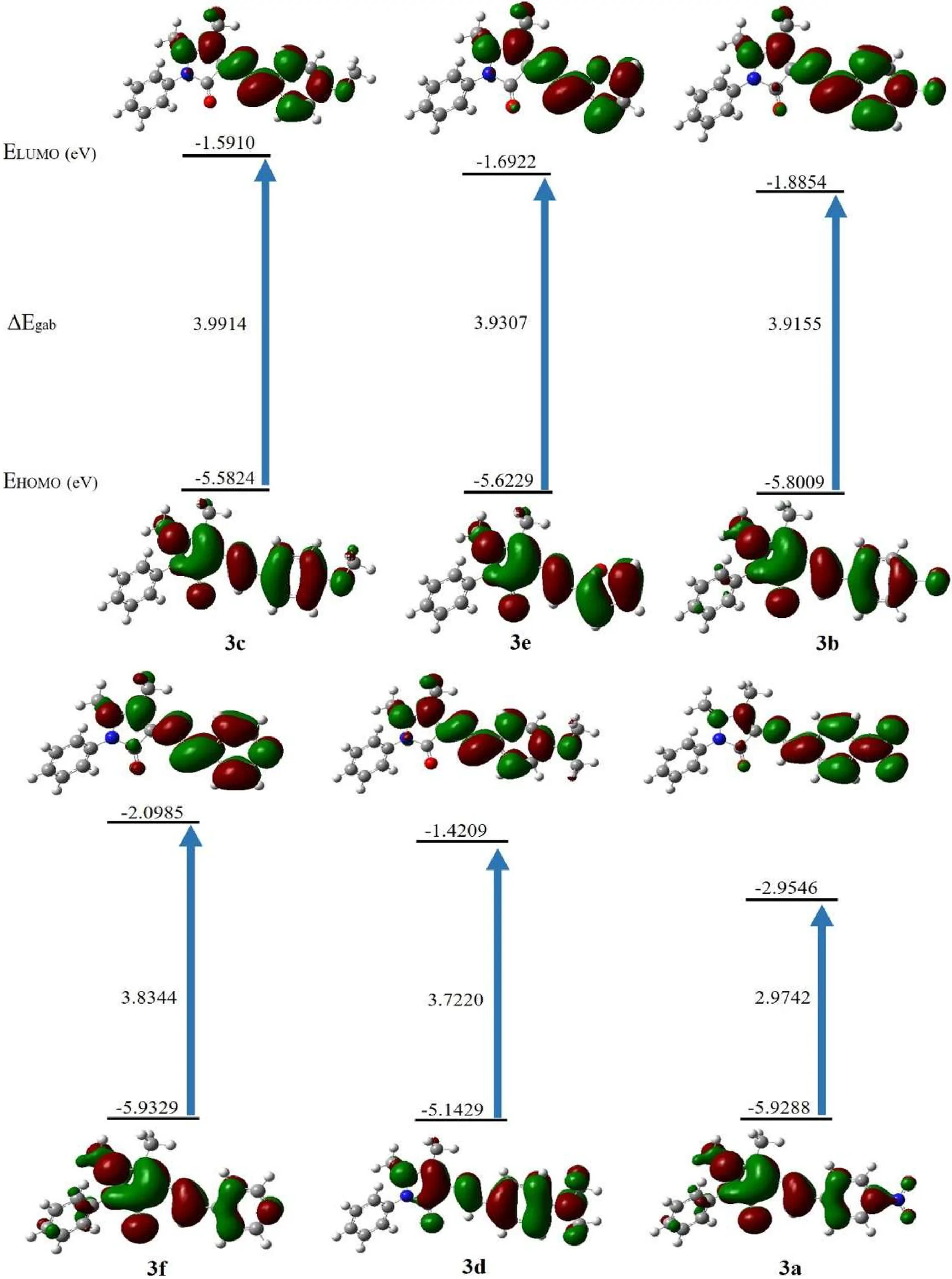
FMOs distribution energy of the Schiff base derivatives (**3a–3f**) at the 6-311G (d,p) level of theory.

**Fig. 8:**
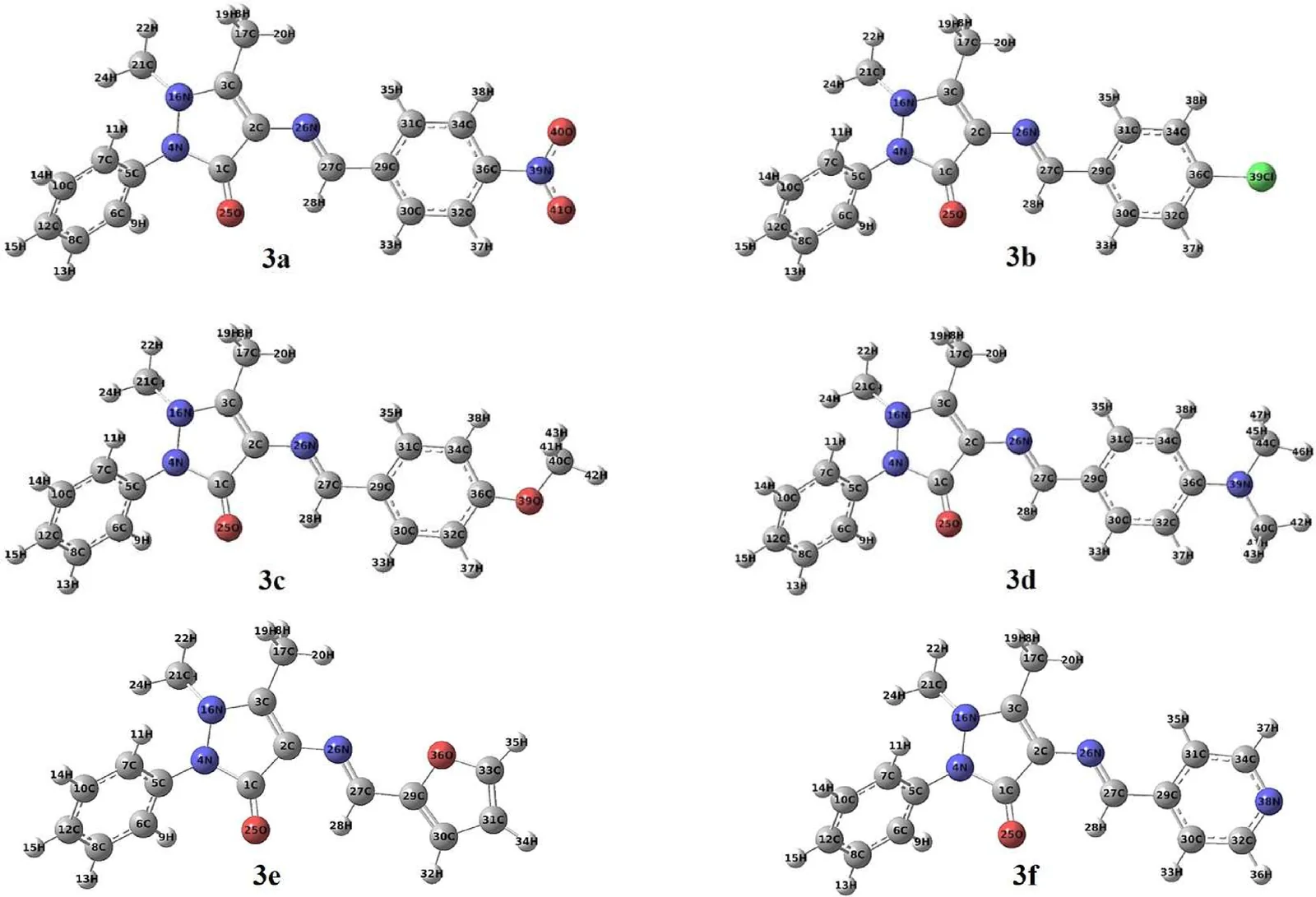
Optimized 3D structures of the Schiff base derivatives (**3a–3f**).

**Fig. 9:**
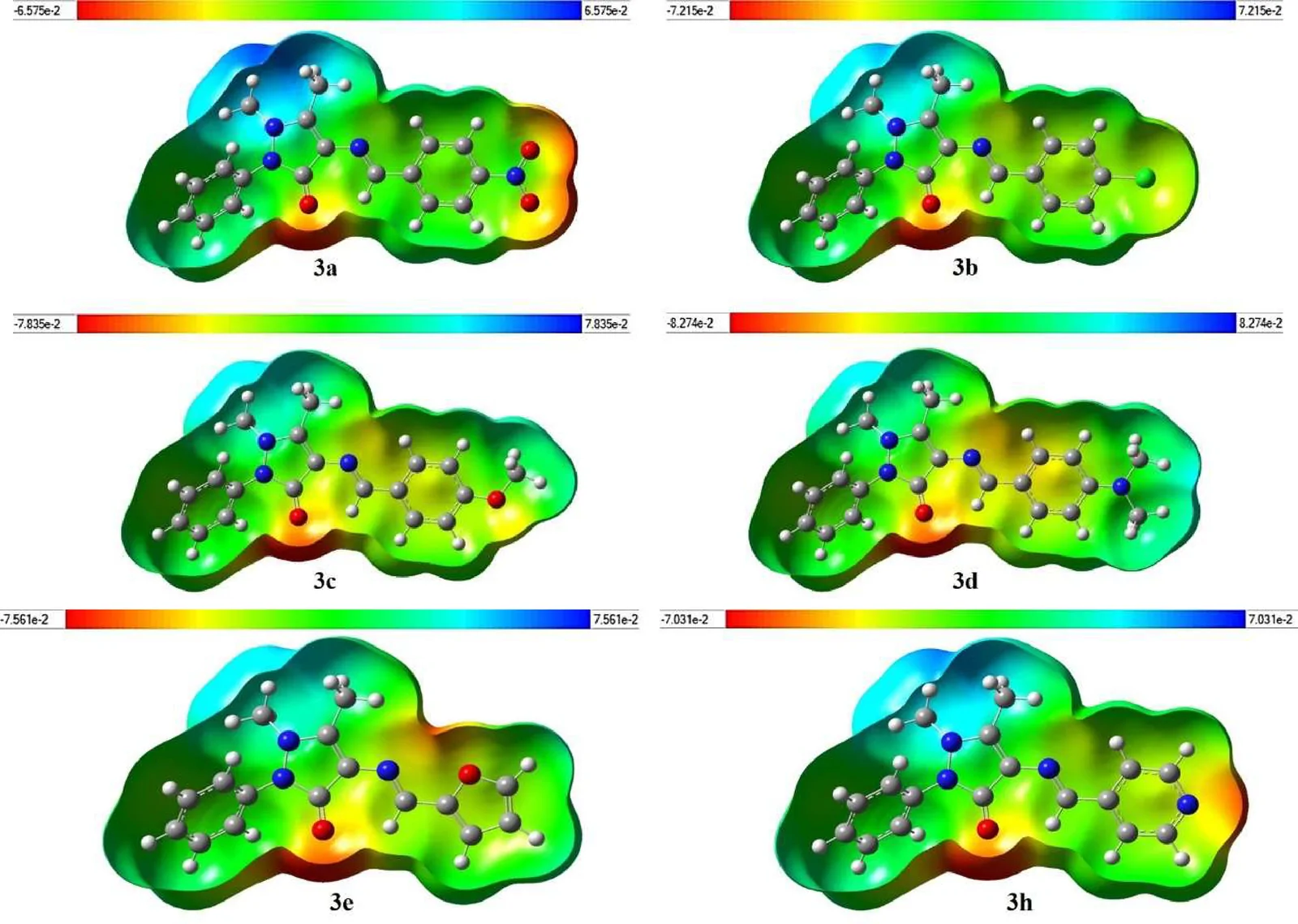
MESP map of the optimized strictures of the Schiff base derivatives (**3a–3f**).

Frontier molecular orbital (FMO) evaluation of the Schiff base derivatives (**3a–3f**), along with their molecular electrostatic potential (MEP) maps, presents a detailed analysis of their electronic structure, charge distribution trends, and potential implications for biological interactions.^51^ The FMO distributions are shown in Fig. 7. The HOMO energies, representing the electron-donating potential, range from −5.93 to −5.14 eV, with compound **3d** showing the lowest negative HOMO energy of −5.14 eV, indicating higher nucleophilicity and increased propensity to transfer electron density to electrophilic protein residues. Although **3a** and **3** **f** have the most negative HOMO energy of −5.93 eV, indicating better internal stabilization and reduced oxidation propensity, they also exhibit relatively low LUMO energies, suggesting a similar trend in their electron-accepting properties. Schiff base derivative **3d**, with a relatively high ELUMO value of −1.42 eV, exhibits moderate reactivity and a relatively lower electron-accepting tendency. These electronic differences are further reflected in the HOMO-LUMO energy gap (ΔE). Compound **3a** has the smallest energy gap (2.97 eV), suggesting comparatively higher chemical reactivity. Meanwhile, compounds **3b**, **3c**, **3d**, and **3** **f** exhibit intermediate ΔE values of 3.72–3.93 eV, showing moderate chemical reactivity. The dipole moment also separates the compounds; **3a** shows the highest polarity of 13.13 Debye, indicating stronger interactions with polar solvents and stronger electrostatic interactions, while compounds including **3d** and **3e** show much lower polarity and possibly more favorable hydrophobic interactions within the hydrophobic regions of the binding pocket. The global reactivity parameters highlight the unique behavior of **3a**, whose lowest chemical hardness η = 1.48 eV and highest softness S = 0.336 eV^−1^ classify it as the most chemically flexible molecule capable of efficient charge redistribution. Its high electrophilicity index (ω = 6.63 eV) and electronegativity (χ = 4.44 eV) suggest a relatively strong electrophilic character and may contribute to interactions with the nucleophilic residues in the enzyme. Geometry optimization was performed at the DFT level using the B3LYP functional and the 6-311G(d,p) basis sets. The optimized 3D structures of the Schiff basis derivatives (**3a–3f**) are shown in Fig. 8. The electron-rich regions of compound 3aMEP analysis supports and enriches the FMO interpretation by revealing spatially resolved charge distributions that control molecular recognition and binding. All compounds show prominent electron-rich regions (red to yellow), mainly located around heteroatoms such as oxygen and nitrogen,^52^ which may represent potential sites for electrophilic attack and hydrogen-bonding interactions as shown in Fig. 9. Compound **3a** exhibits pronounced electron-rich regions distributed across several parts of its molecular surface, consistent with its relatively high dipole moment and softness. These features may facilitate non-covalent interactions, including hydrogen bonding, dipole–dipole interactions, and electrostatic interactions. This apparent polarity indicates that compound **3b** can participate in multiple non-covalent interactions, including hydrogen bonding, dipole-dipole attraction, and electrostatic stabilization. Compounds 3**b** and **3c** show moderately polarized surfaces, with electron-rich regions centered near the carbonyl oxygen, azomethine, and nitrogen atoms, and exhibit consistent expected bond conductivity with their intermediate ΔE values. Compounds **3d**, **3e**, and **3** **f** exhibit distinct electrostatic distributions around their heteroatoms and aromatic rings. These polarized regions may contribute to favorable non-covalent interactions, including hydrogen bonding and π–π interactions, depending on the orientation of the ligand within the binding site. Overall, the FMO and MEP analyses indicate that compound **3a** possesses a relatively small HOMO–LUMO gap, high electrophilicity, and pronounced surface polarization, whereas compounds 3d–3f display comparatively balanced electronic characteristics. Based on their favorable electronic characteristics and the overall experimental findings, compounds **3a**, **3e**, and **3** **f** were selected for in vitro evaluation against HepG2 cells. Compound **3a** exhibited the lowest HOMO–LUMO energy gap, the highest electrophilicity index, and relatively high softness, suggesting comparatively greater chemical reactivity.

Compounds **3e** and **3** **f** showed relatively balanced electronic properties and well-defined charge distributions that may favor interactions with residues within the VEGFR2 active site. These findings support their consideration as potential anticancer candidates.

#### 2.3.2 ADME prediction studies of the synthesized compounds (**3a**–**3f**)

The ADME evaluation of compounds (**3a–3f**), using the SwissADME interface, provides comprehensive information on their physicochemical behavior, pharmacokinetic performance, drug-likeness, and metabolic interaction potential.^53^ Taken together, those parameters help explore the importance of each compound as potential lead candidates for antimicrobial or anticancer drug development. Table 1 summarizes the complete ADME, physicochemical, pharmacokinetic, and metabolic profiles of the studied compounds (**3a–3f**). Physicochemically, all synthesized compounds exhibit molecular weights in the appropriate range for oral drug candidates (<500 Da), which ensures suitable oral bioavailability and compliance with Lipinski's criteria^54^, and log P_O_/W values remain within the recommended range (−0.4 to +5.0), indicating balanced lipophilicity, which aids in membrane permeation without being too hydrophobic. The hydrogen bond acceptor values ranged from 2 to 4, whereas all compounds showed zero hydrogen bond donors, which is also within the permissible range, facilitating passive diffusion across biological barriers. Topological Polar Surface Areas (TPSA) values range from 39.29 to 85.11 Å^2^, which is below the 140 Å^2^ cutoff required for good intestinal absorption and in most cases below the 70 Å^2^ threshold normally associated with blood-brain barrier (BBB) permeability. Pharmacokinetic elements further support these observations. All compounds were predicted to have high gastrointestinal absorption, which is consistent with their TPSA and lipophilicity values. Compound 3a exhibited a relatively balanced lipophilicity and TPSA profile. All compounds were predicted to be BBB-permeant according to the SwissADME model. Although compound **3a** possesses the highest TPSA (85.11 Å^2^), its predicted BBB permeability is likely influenced by the combined effects of multiple molecular descriptors, including lipophilicity, molecular size, and overall molecular structure, rather than TPSA alone. None of the synthesized compounds were predicted to be P-glycoprotein substrates, indicating lower susceptibility to efflux-mediated transport and appropriate intracellular retention. Skin permeation values (log Kp) fall within the typical range of small, moderately lipophilic molecules, suggesting moderate skin permeability. Drug similarity assessment establishes excellent compliance on all Lipinski, Ghose, Veber, Egan, and Muegge filters with zero violations, indicating that the entire collection fits in the physicochemical space of orally active drugs.^55^ Their ESOL-required solubility places all compounds within the “fairly soluble” category, which is often sufficient for oral drug delivery and formulation development. A bioavailability score of 0.55 indicates good oral availability and supports further biochemical evaluation. Metabolic predictions show greater differences between compounds. Many molecules are expected to be inhibitors of leading cytochrome P450 isoenzymes (CYP1A2, CYP2C19 and CYP2C9), especially **3b**, **3d**, **3e,** and **3** **f**, which may additionally affect the metabolic stability in and ability to interact with drugs. Compound **3a** has less inhibitory effects, indicating a metabolically more favorable metabolic profile and possibly a lower risk of toxicity. Neither compound inhibits CYP2D6 or CYP3A4, two primary enzymes related to systemic drug metabolism, reducing the potential for clinically major metabolic interference.

**Table 1:** Comprehensive ADME, physicochemical, pharmacokinetic, drug-like, and metabolic profiles of Schiff base derivatives (**3a–3f**).

Parameters	3a	3b	3c	3d	3e	3f
Physicochemical properties						
Molecular weight (Da)	336.34 g/mol	325.79 g/mol	334.41 g/mol	292.34 g/mol	281.31 g/mol	292.34 g/mol
Log Po/w (MLOGP)	2.13	3.59	2.99	2.03	1.82	2.03
No. of hydrogen bond acceptors	4	2	2	3	3	3
No. of hydrogen bond donors	0	0	0	0	0	0
Molar refractivity	98.77	94.96	104.16	87.75	82.22	87.75
No. of rotatable bonds	4	3	4	3	3	3
TPSA	85.11 Å^2^	39.29 Å^2^	42.53 Å^2^	52.18 Å^2^	52.43 Å^2^	52.18 Å^2^
Pharmacokinetics						
Gastrointestinal (GI) absorption	High	High	High	High	High	High
(BBB) permeant	No	Yes	Yes	Yes	Yes	Yes
P-glycoprotein substrate	No	No	No	No	No	No
Log Kp (skin permeation)	−6.97 cm/s	−6.33 cm/s	−6.75 cm/s	−7.34 cm/s	−6.94 cm/s	−7.34 cm/s
Drug likeness						
Log S (ESOL)	−3.39	−3.94	−3.56	−2.69	−2.91	−2.69
Water solubility class	Moderately soluble	Moderately soluble	Moderately soluble	Moderately soluble	Moderately soluble	Moderately soluble
Lipinski rule	Yes; 0 violation	Yes; 0 violation	Yes; 0 violation	Yes; 0 violation	Yes; 0 violation	Yes; 0 violation
Ghose	Yes	Yes	Yes	Yes	Yes	Yes
Veber	Yes	Yes	Yes	Yes	Yes	Yes
Egan	Yes	Yes	Yes	Yes	Yes	Yes
Muegge	Yes	Yes	Yes	Yes	Yes	Yes
Bioavailability score	0.55	0.55	0.55	0.55	0.55	0.55
Metabolism						
CYP1A2 inhibitor	No	Yes	No	Yes	Yes	Yes
CYP2C19 inhibitor	No	Yes	Yes	Yes	Yes	Yes
CYP2C9 inhibitor	Yes	Yes	Yes	No	Yes	No
CYP2D6 inhibitor	No	No	No	No	No	No
CYP3A4 inhibitor	No	No	No	No	No	No

In summary, the ADME effects indicate that each of the six compounds have drug-like properties, high intestinal absorption, and physicochemical properties favorable for biological activity. Compound 3a stands out of its balanced lipophilicity, standard solubility, high absorption, and minimal CYP inhibition, Compounds **3d**, **3e**, and **3** **f** also show promising ADME profiles with good absorption and mild metabolic interaction potential. The DFT and ADME analyses of the synthesized Schiff bases further support the experimental findings by demonstrating that the derivatives possess favorable electronic and pharmacokinetic characteristics. Similar relationships between HOMO–LUMO energy gap, molecular electrostatic potential, drug-likeness, and biological activity have recently been reported for structurally related Schiff base derivatives, indicating that these computational descriptors provide valuable insight into the structure–property–activity relationship of this class of compounds.^56^

#### 2.3.3 Molecular docking studies of the synthesized compounds (**3a–3f**)

##### 2.3.3.1 Molecular docking of the synthesized compounds (**3a–3f**) to DNA gyrase

DNA gyrase is a type II topoisomerase found in bacteria that is essential for controlling DNA topology during transcription, replication, and chromosomal segregation. It does this by ATP-dependently inserting negative supercoils into DNA.^57^ Because this enzyme is essential for bacterial survival and is absent in higher eukaryotes, it has become an important target in the development of antibacterial agents. In particular, several classes of antibiotics exert their antibacterial activity through inhibition of DNA gyrase, highlighting its importance as a promising target for the discovery of new antimicrobial compounds. The crystal structure provides detailed structural information about the 4DUH active site and key residues involved in ligand binding.^58^

The co-crystallized ligand was re-docked into the active site using the same docking parameters in order to verify the docking procedure. The re-docking experiment produced a binding energy of −8.98 kcal·mol^−1^ and an RMSD value of 1.121 Å between the predicted and crystallographic poses. Since RMSD values below 2.0 Å are generally considered indicative of a reliable docking methodology, these results confirm the validity of the applied docking protocol.

**Fig. 10:**
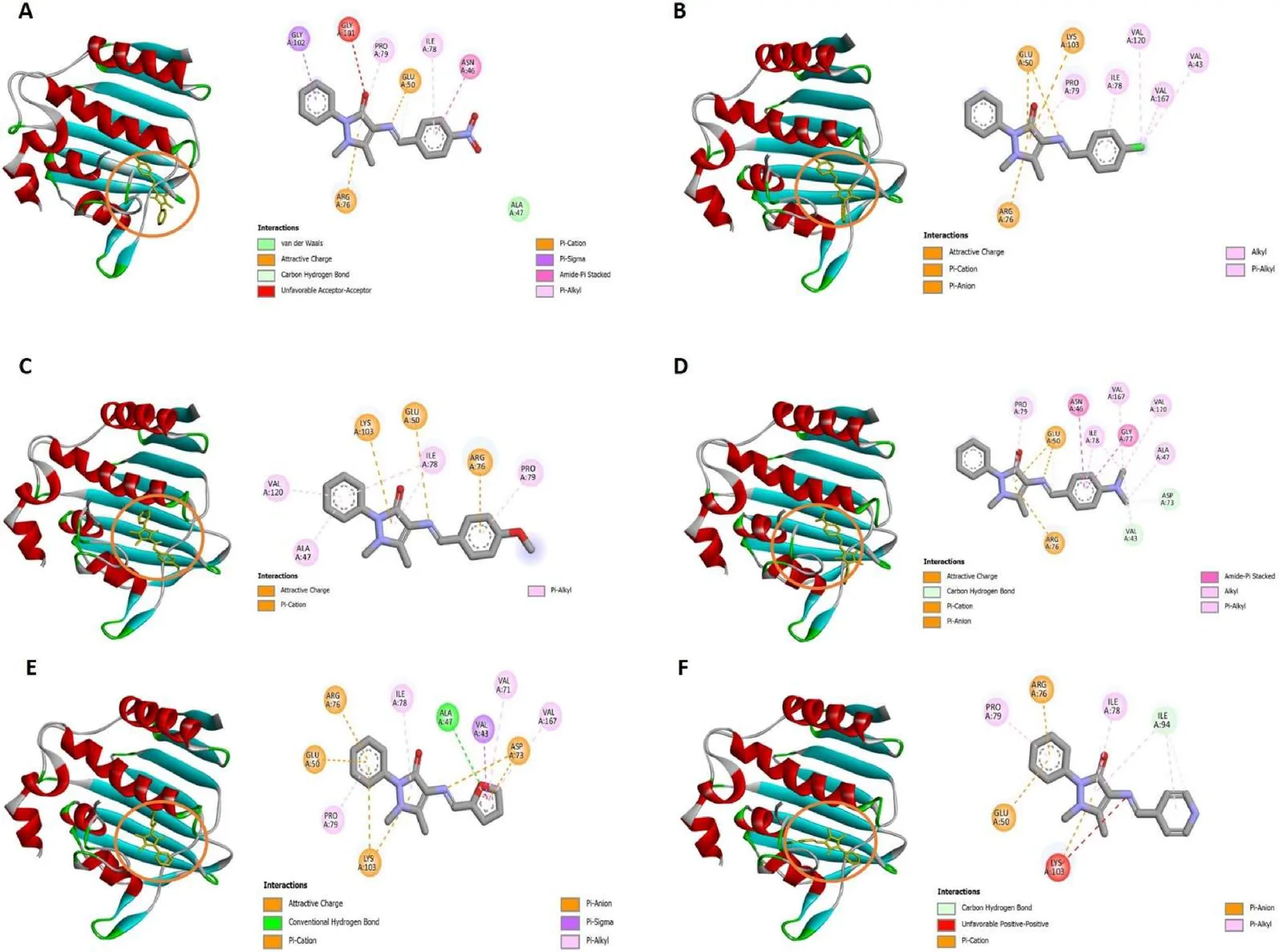
Binding of the Schiff base derivatives (3a-f) inside the DNA gyrase. **A. 3a, B. 3b, C. 3c, D. 3d, E. 3e, F. 3** **f.**

The synthesized derivatives (**3a–3f**) were docked inside the active site of DNA gyrase (PDB ID: 4DUH) after removal of the co-crystallized ligand to investigate their binding affinity and mode of interaction toward the enzyme (Fig. 10). Molecular docking results revealed that the synthesized compounds (**3a–3f**) interact with the active site of the target enzyme through various non-covalent interactions including hydrogen bonds.

Compound **3e** exhibited the strongest predicted binding affinity, with a docking score of - 8.2 kcal/mol, while compounds **3** **f** and **3c** showed binding energies of −7.7 and − 7.3 kcal/mol, respectively. Other synthesized compounds **3a**, **3b** and **3d** showed similar binding energy of about −6.9 kcal/mol.

Further analysis of the docking positions revealed that the synthesized compounds interact with the active site residues through various non-covalent interactions including hydrogen bonds, electrostatic interactions and hydrophobic contacts. Compounds **3a** and **3b** interact primarily through electrostatic interactions involving residues such as Arg76, Glu50 and Lys103, in addition to hydrophobic π-acyl contacts. In particular, compound **3c** interacted primarily through π-cation interactions with Arg76 and Lys103 and an attractive charge interaction with Glu50, while compound **3d** showed amide π-stacking with Pro79 and additional electrostatic interactions with Arg76 and Glu50. Compound **3e** showed a favorable binding pattern via hydrogen bonding with Ala47 with several electrostatic interactions, while compound **3** **f** formed π-cation and π-anion interactions with Arg76 and Glu50; however, an unfavorable positive–positive interaction was observed with Lys103. Overall, hydrogen bonding, electrostatic forces, and hydrophobic interactions play important roles in stabilizing the ligand-protein complex in the enzyme binding pocket.

##### 2.3.3.2 Molecular docking of the synthesized compounds (**3a–3f**) to VEGFR2 kinase

**Fig. 11:**
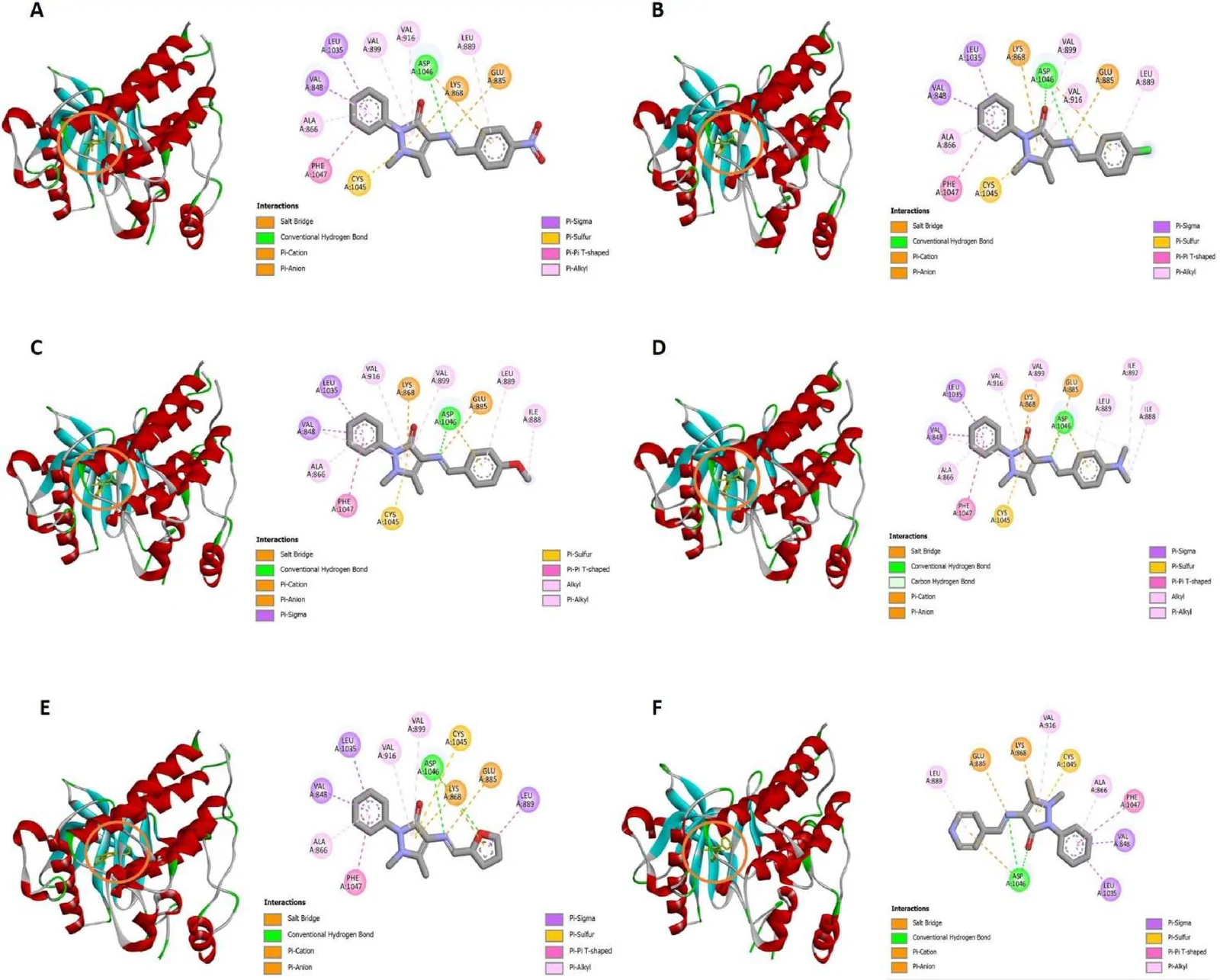
Binding of the Schiff base derivatives (3a-f) inside the VEGFR2 kinase. **A. 3a, B. 3b, C. 3c, D. 3d, E. 3e, F. 3** **f.**

Molecular docking simulations were performed to investigate the binding mode of the synthesized Schiff base derivatives (**3a–3f**) within the catalytic pocket of Vascular Endothelial Growth Factor Receptor 2 (Fig. 11) using the crystal structure of VEGFR2 (PDB ID: 3VHE).^59^ VEGFR2 is a key tyrosine kinase receptor that plays a crucial role in the regulation of angiogenesis, endothelial cell proliferation, migration, and survival. Dysregulation of VEGFR2 signaling has been strongly associated with tumor progression and metastasis, making this receptor an important therapeutic target for anticancer drug development.^60^

The co-crystallized ligand was re-docked into the active site using the same docking conditions in order to verify the docking procedure. A binding energy of −12.5 kcal.mol^−1^ and an RMSD value of 0.841 Å between the predicted and crystallographic ligand poses were obtained from the re-docking experiment. These results validate the docking protocol, as RMSD values below 2.0 Å are generally considered indicative of a reliable docking procedure. The docking outcomes showed that all the synthesized compounds fit well within the ATP-binding pocket of VEGFR2 and anchored several stabilizing interactions with key residues lining the catalytic cavity. Notably, most derivatives formed a conventional hydrogen bond with Asp1046, a residue of the conserved DFG motif that plays an important role in kinase activity and ligand stabilization. Furthermore, electrostatic interactions with Lys868 and Glu885 were frequently observed, contributing significantly to stabilization through π–cation and π–anion interactions. In addition, several hydrophobic interactions were observed within the binding pocket, including π–π stacking and π–alkyl interactions with Phe1047, Val848, Leu1035, Val899, Val916, and Leu889, which form the hydrophobic environment in the ATP-binding cavity.

Additional π–sulfur interactions with Cys1045 were identified, further contributing to the stabilization of the ligand within the binding site. In terms of binding affinity, compounds **3a**, **3e**, and **3** **f** showed the most favorable docking scores with binding energies of −10.3 kcal.mol^−1^. Compounds **3c**, **3b**, and **3d** showed slightly lower binding energies of −10, −9.9, and − 9.4 kcal.mol^−1^, respectively. Among the studied derivatives, compounds **3e** and **3** **f** showed the most favorable interaction profiles, forming a pair of hydrogen bonds and strong electrostatic interactions with key residues in the active site. Overall, the determined interaction manners are consistent with those for clinically used VEGFR2 kinase inhibitors such as Sorafenib and Sunitinib, which exert their inhibitory activity by occupying the ATP-binding pocket and forming multiple hydrogen bonds and hydrophobic interactions within the kinase domain.^61^ Therefore, the ability of the synthesized Schiff base derivatives to establish similar interactions with important residues containing Asp1046 and Lys868 suggests that those compounds may also act as VEGFR2 inhibitors capable of interfering with ATP binding and suppressing angiogenesis -related signaling. Vascular endothelial growth factor receptor-2 (VEGFR2) is a key tyrosine kinase receptor that plays a crucial role in the regulation of angiogenesis, endothelial cell proliferation, migration, and survival. The favorable molecular docking interactions of the synthesized Schiff base with VEGFR2 observed in the present study support the experimental cytotoxicity results and may help to explain that these compounds may interfere with signaling pathways involved in tumor growth and angiogenesis.^62^ Although the synthesized Schiff bases compounds (3a–3f) exhibited potent antibacterial, antioxidant, and anticancer activities, the current study was limited to in vitro and computational investigations. In particular, MIC/MBC values were not determined, the cytotoxicity evaluation was limited to the HepG2 and Vero cell lines, and the underlying biological mechanisms were not experimentally validated. Future works should focus on the synthesis of additional Schiff base derivatives and mechanistic investigations to achieve broader structure–property–activity relationships (SPAR), and in vivo evaluation of the most promising derivatives. The structural modification and optimization of bioactive molecules remain important strategies for developing more effective antimicrobial and anticancer agents.^63^

## 3 Materials and methods

### 3.1 Chemistry

#### 3.1.1 General

All solvents and starting materials were obtained from commercial suppliers, including Merck Group and Sigma-Aldrich, and were similarly used without purification. All melting points were measured using an electrothermal equipment (Stuart SMP-30) and are reported without correction. FT-IR spectra were recorded on a Bruker ALPHA II FTIR spectrometer equipped with an ATR module over the spectral range of 4000–400 cm^−1^.UV–Vis absorption spectra are recorded using Pye-Unicam 8800. ^1^H NMR spectra were acquired on a 300 MHz spectrometer (Bruker BioSpin GmbH), with chemical shifts referring to the residual solvent signal of DMSO‑*d*_6_ (*δ* = 2.50 ppm). GC–MS analyses were performed using an Agilent 7890B Gas Chromatograph coupled with an Agilent 5977B Mass Selective Detector (MSD).

#### 3.1.2 Synthesis of Schiff bases (3a–3f)

Schiff base was synthesized by the condensation reaction between 4-amino-1,5-dimethyl-2-phenylpyrazol-3-one (4-AAP) and various aromatic aldehydes. An anhydrous ethanol solution (10 mL) containing 4-amino-1,5-dimethyl-2-phenylpyrazol-3-one (0.203 g, 0.001 mol) was mixed with another anhydrous ethanol solution (10 mL) of the corresponding *p*-substituted benzaldehyde (**2a-d**), furan-2-carbaldehyde (**2e**), and pyridine-4-carbaldehyde (**2f**) (0.001 mol). The reaction mixture was refluxed at 80 °C for five to seven hours under atmospheric conditions in a round-bottom flask fitted with a reflux condenser. The completion of the reaction was monitored by TLC using Benzene: Ethyl acetate solvent system. Following completion, the reaction mixture was allowed to cool to room temperature. The precipitate that resulted was then filtered and recrystallized from cold ethanol to obtain pure Schiff base in moderate to high yield (71–86%).^16^

1,5-dimethyl-4-((4-nitrobenzylidene)amino)-2-phenyl-1,2-dihydro-3H-pyrazol-3-one (**3a**).

Orange solid, yield 86%, m.p. 223–225 °C; FT-IR (cm^−1^), 3157, 3136 ν(C—H aromatic), 2959 ν(C—H aliphatic), 2824 ν(HC=N azomethine),1638 ν(C=O antipyrine ring), 1596 ν(C=N), 1571 ν(C=C aromatic), 1550 and 1335 νas(NO₂) and νs(NO₂), respectively. ^1^H NMR (300 MHz, DMSO‑*d*_6_) *δ* (ppm), 9.66 (s, 1H, –N=CH), 8.27 (d, *J* = 9 Hz, 2H, Ar—H), 8.04 (d, *J* = 9 Hz, 2H, Ar—H), 7.57–7.52 (m, 2H, Ar—H), 7.43–7.37 (m, 3H, Ar—H), 3.30 (s, 3H, –N–CH_3_), 3.25 (s, 3H, =C–CH_3_). MS (NCI): Calculated for C₁₈H₁₆N₄O₃ (M^+^), 336.3; found *m*/*z* 336.3, Rf = 0.76 (1,2, Benzene, Ethyl acetate).

4-((4-chlorobenzylidene)amino)-1,5-dimethyl-2-phenyl-1,2-dihydro-3H-pyrazol-3-one (**3b**).

Pale yellow solid, yield 77%, m.p. 253–254 °C; FT-IR (cm^−1^), 3059, 3034 ν(C—H aromatic), 2937 ν(C—H aliphatic), 2919 ν(HC=N azomethine), 1646 ν(C=O antipyrine ring), 1591 ν(C=N), 1570 ν(C=C aromatic), 828 ν(C—Cl). ^1^H NMR (300 MHz, DMSO‑*d*_6_) *δ* (ppm), 9.56 (s, 1H, –N=CH), 7.82 (d, *J* = 8.7 Hz, 2H, Ar—H), 7.36–7.56 (m, 7H, Ar—H), 3.19 (s, 3H, –N–CH_3_), 2.46 (s, 3H, =C–CH_3_). MS (NCI): Calculated for C₁₈H₁₆ClN₃O (M^+^), *m*/*z* 325.8; found *m*/*z* 325.3., Rf = 0.83 (1,2, Benzene, Ethyl acetate).

4-((4-methoxybenzylidene)amino)-1,5-dimethyl-2-phenyl-1,2-dihydro-3H-pyrazol-3-one (**3c**).

Light yellow solid, yield 75%, m.p. 169–170 °C; FT-IR (cm^−1^), 3050, 3033 ν(C—H aromatic), 2932 ν(C—H aliphatic), 2832 ν(HC=N azomethine), 1642 ν(C=O antipyrine ring), 1591 ν(C=N), 1506 ν(C=C aromatic). ^1^H NMR (300 MHz, DMSO‑*d*_6_) *δ* (ppm), 9.52 (s, 1H, –N=CH), 7.75 (d, *J* = 8.7 Hz, 2H, Ar—H), 7.55–7.50 (m, 2H, Ar—H), 7.39–7.33 (m, 3H, Ar—H), 7.01 (d, *J* = 8.7 Hz, 2H, Ar—H), 3.81 (s, 3H, –OCH_3_), 3.14 (s, 3H, –N–CH_3_), 2.43 (s, 3H, =C–CH_3_). MS (NCI): Calculated for C₁₉H₁₉N₃O₂ (M^+^), *m*/*z* 321.3; found *m*/*z* 321.3, Rf = 0.80 (1,2, Benzene, Ethyl acetate).

4-((4-(dimethylamino)benzylidene)amino)-1,5-dimethyl-2-phenyl-1,2-dihydro-3H-pyrazol-3-one (**3d**).

Yellow solid, yield 78%, m.p. 219–221 °C; FT-IR (cm^−1^), 3010, 3025 ν(C—H aromatic), 2958 ν(C—H aliphatic), 2891 ν(HC=N azomethine), 1644 ν(C=O antipyrine ring), 1609 ν(C=N), 1578 ν(C=C aromatic). ^1^H NMR (300 MHz, DMSO‑*d*_6_) *δ* (ppm), 9.43 (s, 1H, –N=CH), 7.62 (d, *J* = 9 Hz, 2H, Ar—H), 7.54–7.49 (m, 2H, Ar—H), 7.39–7.32 (m, 3H, Ar—H), 6.75 (d, *J* = 9 Hz, 2H, Ar—H), 3.10 (s, 3H, –N–CH_3_), 2.98 (s, 6H, –N–C_2_H_6_), 2.41 (s, 3H, =C–CH_3_). MS (NCI): Calculated for C₂₀H₂₂N₄O (M^+^), *m*/*z* 334.4; found *m*/*z* 334.3, Rf = 0.81 (1,2, Benzene, Ethyl acetate).

4-((furan-2-ylmethylene)amino)-1,5-dimethyl-2-phenyl-1,2-dihydro-3H-pyrazol-3-one (**3e**).

Dark yellow crystals, yield 76%, m.p. 209–210 °C; FT-IR (cm^−1^), 3110, 3045 ν(C—H aromatic), 2958 ν(C—H aliphatic), 2924 ν(HC=N azomethine), 1642 ν(C=O antipyrine ring), 1590 ν(C=N), 1545 ν(C=C aromatic). ^1^H NMR (300 MHz, DMSO‑*d*_6_) *δ* (ppm), 9.44 (s, 1H, –N=CH), 7.84 (d, *J* = 1.5 Hz, 1H, –O-CH furan ring), 7.53 (dd, *J* = 7.8, 7.8 Hz, 2H, Ar—H), 7.39–7.35 (m, 3H, Ar—H), 6.95 (d, *J* = 3.9 Hz, 1H, Ar—H furane), 6.63 (dd, *J* = 3.6, 1.8 Hz, 1H, Ar—H furane), 3.16 (s, 3H, –N–CH_3_), 2.40 (s, 3H, =C–CH_3_). MS (NCI): Calculated for C₁₆H₁₅N₃O₂ (M^+^), *m*/*z* 281.3; found *m*/*z* 281.2, Rf = 0.67(1,2, Benzene, Ethyl acetate).

1,5-dimethyl-2-phenyl-4-((pyridin-4-ylmethylene)amino)-1,2-dihydro-3H-pyrazol-3-one (**3f**).

Yellow solid, yield 71%, m.p. 189–191 °C; FT-IR (cm^−1^), 3035 ν(C—H aromatic), 2955 ν(C—H aliphatic), 2925 ν(HC=N azomethine),1639 ν(C=O antipyrine ring), 1595 ν(C=N), 1552 ν(C=C aromatic). ^1^H NMR (300 MHz, DMSO‑*d*_6_) *δ* (ppm), 9.56 (s, 1H, –N=CH), 8.65 (d, *J* = 6.3 Hz, 2H, Ar—H pyridine), 7.74 (d, *J* = 6.3 Hz, 2H, Ar—H pyridine), 7.56 (t, *J* = 7.5 Hz, 2H Ar—H), 7.44–7.38 (m, 3H Ar—H), 3.32 (s, 3H, –N–CH_3_), 3.25 (s, 3H, =C–CH_3_). MS (NCI): Calculated for C₁₇H₁₆N₄O (M^+^), *m*/*z* 292.34; found *m*/*z* 292.2, Rf = 0.71(1,2, Benzene: Ethyl acetate).

### 3.2 Biology

#### 3.2.1 Antibacterial activity of the Schiff base derivatives (**3a–3f**)

Agar well-diffusion assay was used to assess the antimicrobial properties of the synthesized Schiff base derivatives (**3a–3f**) against a variety of bacteria including *Acinetobacter baumannii* (ATCC 19606) (Gram-negative) and Gram-positive bacteria such as *Staphylococcus aureus* (ATCC 25923). These isolates came from the Department of Biology, College of Science, Mustansiriyah University in Baghdad, Iraq. Briefly, 100 mL of deionized water was used to dissolve 3.8 g of Mueller-Hinton agar. The resulting medium was autoclaved for sterilization. Indian Pharmacopoeia was followed to carry out the sterilization cycle. Nutrient agar (20 mL) was then added to petri plates which were sterilized. Bacterial strains were extracted from stock bacterial cultures using a sterile loop. Mueller-Hinton agar was used to deliver the bacterial inoculum. The agar medium was punctured with wells (6 mm diameter) using a sterile tip.^64^ The wells were filled with test Schiff base derivatives at concentrations of 500, 250, 125, 62.5, and 31.25 μg/mL in DMSO. The plates containing the test compounds and corresponding microorganisms were incubated at 37 °C for 24 h. A standard concentration of meropenem (10 μg/disc) was used as a positive control to validate the antibacterial assay, while test compounds were evaluated at higher concentrations to assess their antibacterial potential. Zones of inhibition were used to support the antibacterial effect of the test compounds. Inhibition zones were measured in millimeters, using a ruler and taking into account the average value of each well.

#### 3.2.2 Antioxidant activity of the Schiff base derivatives (**3a–3f**)

The evaluation of free radical scavenging activity was done in accordance with Akar et al..^65^ Five concentrations of the compounds (**3a–3f**) were assessed: 31.25, 62.5, 125, 250, and 500 μg/mL in DMSO. The doses described above were combined with a methanolic solution of DPPH (2,2-diphenyl-1-picrylhydrazyl) (1 mL, 0.4 μM). The test sample (3.0 mL) was then diluted with 1 mL of methanol prior to analysis. After 60 min, a Pye-Unicam 8800 UV–visible spectrophotometer was used to evaluate the reducing activity of DPPH free radicals and record the absorbance at 517 nm. Ascorbic acid (Vitamin C) was used as the positive control at a concentration of 5 μg/mL. The stock solution was prepared by dissolving 0.5 mg of ascorbic acid in 100 mL of a 1:1 (*v*/v) ethanol–distilled water mixture. Eq. 1 was used to determine the free radical antioxidant activity.

(1) $$\text{DPPH Scavenging}\;\left(\%\right)=\left(A_{C}–A_{S}\right)/A_{C}\;x\;100$$

Ac: is the absorption of control, and the As: is the absorption of the tested compounds.

#### 3.2.3 Cytotoxicity (MTT assay)

In order to conduct this experiment, 1 × 10^4^ cells were plated in each well of 96-well plates, and the cells were allowed to adhere to the substrate at 37 °C in a humidified 5% CO_2_ incubator. HepG2 cells (passages 5–15) and Vero cells (passages 5–20) were used throughout the experiments. Different concentrations of **3a**, **3e**, and **3** **f** compounds (10, 25, 50, 100, 250, and 500 μg/mL) were applied to cells that had grown to 70–85% confluence. For 48 h, the treated and control cells were kept in an incubator at 37 °C with 5% CO_2_. Following incubation, the MTT (3-(4, 5-dimethylthiazol-2-yl)-2, 5-diphenyl-tetrazolium bromide) assay was used to assess the cell viability in accordance with the standard test methodology.^66^ The cytotoxic activity was calculated using Eq. 2.

(2) $$\text{Cytotoxic Activity}\;\left(\%\right)=100-\text{Cell Viability}\;\left(\%\right)$$

### 3.3 In silico studies

#### 3.3.1 Global reactivity descriptors and molecular electrostatic potential (MEP)

Quantum chemical calculations for Schiff base derivatives (**3a–3f**) were performed using the Gaussian 09 Revision A.02 software package.^67^ The B3LYP functional and the 6-311G (d,p) basis set were used to optimize geometry at the DFT level^68^.^69^ All structures were fully optimized without symmetry constraints, and the absence of imaginary frequencies confirmed that each optimized geometry corresponds to a true minimum. Solvent effects were added via the polarizable continuum model (PCM) with ethanol. From the optimized structures, frontier molecular orbital energies E_HOMO_ and E_LUMO_, energy difference (ΔE = E_LUMO_ – E_HOMO_), total electronic energy (E_total_) and dipole moment (μ) were calculated. Global reactivity descriptors were obtained according to frontier molecular orbital theory, including ionization potential (I = -E_HOMO_), electron affinity (A = -E_LUMO_), nucleophilicity (N = E_HOMO_ (compound) - E_HOMO_ (TCE)) (where tetracyanoethylene, TCE is a reference electrophile with E_HOMO_ TCE = −9.12 eV). Chemical hardness (η = (I-A)/2), chemical softness (S = 1/2η), electronegativity (χ = (I + A)/2), chemical potential (μ = −χ), and electrophilicity index (ω = μ^2^ / 2η). These parameters were used to evaluate the electronic stability and predicted biological reactivity of the compounds.^70^ Molecular electrostatic potential (MEP) maps were calculated using Gaussian 09 Revision A.02 and visualized with GaussView 5.0 software at the same theoretical level, with the electrostatic potential projected onto the electron density surface at an isovalue of 0.001 au.^71^ The resulting color-coded surfaces reveal electron-rich regions (red) susceptible to electrophilic attack and electron-deficient regions (blue) susceptible to nucleophilic interactions, providing further insight into the active sites of the compounds studied.

#### 3.3.2 ADME prediction studies

The in silico pharmacokinetic and drug-like properties of the Schiff base compounds (**3a–3f**) were investigated using the freely available SwissADME web tool https://www.swissadme.ch established by the Swiss Institute of Bioinformatics.^72^ This platform facilitates accurate prediction of overall drug-like profiles, as well as key physicochemical parameters, absorption, distribution, metabolism and excretion (ADME) properties. For each compound, the SMILES notation was created from optimized molecular structures obtained using the Gaussian 09 program on the B3LYP/6-311G (d,p) basis set.^73^ These SMILES chains were then sent separately to the SwissADME server. The analysis provided a comprehensive dataset of parameters, such as molecular weight (MW), number of hydrogen bond acceptors (HBA), number of hydrogen bond donors (HBD), number of rotatable bonds, topological polar surface area (TPSA), partition coefficient (Log P_O_/w) and molar refractivity. In addition, physicochemical properties including gastrointestinal (GI) absorption, blood-brain barrier (BBB) permeability, skin permeability (Log Kp) and P-glycoprotein (P-gp) substrate prediction were obtained. Drug similarity was assessed according to Lipinski's rule of five, as well as other predictive filters including the Ghose, Veber, Egan and Muegge criteria. Furthermore, a bioavailability score was calculated for each compound to estimate the probability of achieving satisfactory oral bioavailability. The metabolic stability of the compounds was assessed through assessment of potential inhibition of key cytochrome P450 isoenzymes (CYP1A2, CYP2C19, CYP2C9, CYP2D6 and CYP3A4). The inclusive ADME analysis provided a theoretical basis for assessing drug similarity, pharmacokinetic effects and metabolic conformity of the synthesized compounds. The data obtained were correlated with electronic parameters obtained from quantum chemical calculations to establish a structure–property–activity relationship (SPAR) model supporting their potential biological activity.

#### 3.3.3 Molecular docking studies

In this study, molecular docking was employed to investigate the potential interactions between the synthesized Schiff base derivatives (**3a–3f**) and key enzymes. To assess the antibacterial and anticancer properties of these drugs, molecular docking was done against key microbial and cancer enzymes. The target enzymes; DNA gyrase (PDB ID: 4DUH) from *Escherichia coli*^23^ and VEGFR2 kinase (PDB ID: 3VHE) from *Homo sapiens.*^24^ The three-dimensional structures of these enzymes were obtained from the RCSB Protein Data Bank (https://www.rcsb.org/ accessed on March 17, 2026) as shown in Table 2. Prior to docking, non-essential components such as water molecules and co-crystallized ligands were eliminated using BIOVIA Discovery Studio Visualizer 2024, the 3D structures of the Schiff base derivatives (**3a–3f**) were constructed in .sdf format using ChemDraw 19.1 and converted to .pdb format using Open Babel 3.1.1 after energy minimization. Docking simulations of the target enzymes DNA gyrase and VEGFR2 kinase were conducted using the PyRx AutoDock Vina 0.8.^74^ The grid centre coordinates were X = 3.221, Y = 2.506, Z = 36.713 with size equal to 32.435, 10.573, and 26.144 for the DNA gyrase, while the dimension applied for the VEGFR2 kinase target was X = −26.025, Y = −1.696, Z = −10.247 with size equal to 34.024, 23.726, and 11.384 as the grid spacing and the affinity (kcal/mol) value was calculated. The best scoring positions for each compound were analyzed and visualized using BIOVIA Discovery Studio 2024, focusing on hydrogen bonding, hydrophobic interactions, π–π stacking and other important contact types. To validate the docking protocol, a redocking procedure was performed by extracting the native co-crystallized ligands from the active sites of both target enzymes DNA gyrase (PDB ID: 4DUH) and VEGFR2 kinase (PDB ID: 3VHE) and re-docking them into their respective binding pockets using the same docking settings and grid described previously. The accuracy of the docking protocol was determined by calculating the root mean square deviation (RMSD) between the redocked and native crystallographic ligand conformations, RMSD was calculated using the online tool DockRMSD.^75^

**Table 2:** Details of the 3D crystal structures (PDB ID: 4DUH) of the DNA gyrase B and (PDB ID: 3VHE) of the VEGFR2 kinase.

Target Enzyme 3D Structure	3D Structure
Code (ID): 4DUH Method: X-ray Diffraction Resolution: 1.50 Å Number of residues: 4*40* Organisms: *Escherichia coli*	
Code (ID): *3*VHE Method: X-ray Diffraction Resolution: 1.55 Å Number of residues: 359 Organisms: *Homo sapiens*	

## 4 Conclusions

In this study, a series of six Schiff base derivatives (3a–3f) derived from 4-aminoantipyrine were synthesized and characterized using several spectroscopic techniques. The synthesized compounds showed promising antibacterial, antioxidant, and cytotoxic activities, with their biological performance strongly affected by the electronic nature of the substituents. Among the derivatives investigated, compound **3a** demonstrated the strongest antibacterial activity against both *Staphylococcus aureus* and *Acinetobacter baumannii*, whereas compound **3d** exhibited the highest antioxidant activity in the DPPH radical scavenging assay. Furthermore, compound **3** **f** exhibited the greatest cytotoxic activity against HepG2 cells while maintaining low toxicity toward normal Vero cells, indicating a favorable selectivity profile. The computational investigations were in good agreement with the experimental results. DFT and molecular electrostatic potential (MEP) analyses provided valuable insights into the electronic properties and chemical reactivity of the Schiff base derivatives, while ADME prediction suggested favorable pharmacokinetic, physicochemical, and drug-like characteristics. Molecular docking approached further demonstrated favorable binding interactions with the binding sites of DNA gyrase and VEGFR2, providing supporting evidence for the observed antibacterial and cytotoxic activities. Furthermore, compound **3e** exhibited the strongest binding affinity toward DNA gyrase, whereas compounds **3a**, **3e**, and **3** **f** showed the most favorable binding affinities toward VEGFR2. The combined experimental and computational findings support the potential of these 4-aminoantipyrine-based Schiff base derivatives as promising multifunctional scaffolds for the development of new antibacterial, antioxidant, and anticancer agents. Further structural optimization together with detailed mechanistic studies and in vivo assay are warranted to fully explore their therapeutic potential.

## CRediT authorship contribution statement

**Mohammed I. Sultan:** Writing – original draft, Methodology, Investigation, Data curation. **Muthanna C. Urabee:** Visualization, Validation, Methodology, Formal analysis. **Ahmed H. Sadiq:** Software, Resources, Project administration, Formal analysis, Data curation. **Ahmed Mutanabbi Abdula:** Writing – review & editing, Validation, Supervision, Methodology, Conceptualization.

## Declaration of competing interest

The authors declare that they have no known competing financial interests or personal relationships that could have appeared to influence the work reported in this paper.
