# Supplementary material for: Synthesis, characterization, and integrated in silico studies of 4-Aminoantipyrine-derived Schiff bases as promising antimicrobial, antioxidant, and cytotoxic agents: DFT, ADME, and molecular docking studies
Source: J Genet Eng Biotechnol. 2026 Sep 10;24(4):100814. doi: 10.1016/j.jgeb.2026.100814 (PMC13580057; doi:10.1016/j.jgeb.2026.100814)
Supplement: Supplementary file 1 [file mmc1.docx]

Supplementary Material

for

**Synthesis, Characterization, and Integrated In Silico studies of 4-Aminoantipyrine-Derived Schiff Bases as Promising Antimicrobial, Antioxidant, and Cytotoxic agents: DFT, ADME, and Molecular Docking Studies**

Mohammed I. Sultan^1^, Muthanna C. Urabee^2^, Ahmed H. Sadiq^3^**,** Ahmed Mutanabbi Abdula^4^

^1^ Quality Assurance & Performance Evaluation Department, Mustansiriyah University, Baghdad, Iraq.

^2,3^ Environmental Research Center, Technology University, Baghdad, Iraq.

^4^Department of Chemistry, College of Science, Mustansiriyah University, Baghdad P.O. Box 14022, Iraq; ahm.chem@uomustansiriyah.edu.iq (A.M.A.)

***Correspondence:** ahm.chem@uomustansiriyah.edu.iq (A.M.A.) Tel.: +964-7808838128

**Table of contents**

| Contents | Page |
| --- | --- |
| Figure S1. FT-IR spectrum of compound 3a. | S3 |
| Figure S2. FT-IR spectrum of compound 3b. | S3 |
| Figure S3. FT-IR spectrum of compound 3c. | S4 |
| Figure S4. FT-IR spectrum of compound 3d. | S4 |
| Figure S5. FT-IR spectrum of compound 3e. | S5 |
| Figure S6. FT-IR spectrum of compound 3f. | S5 |
| Figure S7. ^1^H-NMR spectrum of compound 3a in DMSO-*d*_6_. | S6 |
| Figure S8. ^1^H-NMR spectrum of compound 3b in DMSO-*d*_6_. | S7 |
| Figure S9. ^1^H-NMR spectrum of compound 3c in DMSO-*d*_6_. | S8 |
| Figure S10. ^1^H-NMR spectrum of compound 3d in DMSO-*d*_6_. | S9 |
| Figure S11. ^1^H-NMR spectrum of compound 3e in DMSO-*d*_6_. | S10 |
| Figure S12. ^1^H-NMR spectrum of compound 3f in DMSO-*d*_6_. | S11 |
| Figure S13. GC-MS (NCI) of compound 3a. | S12 |
| Figure S14. GC-MS (NCI) of compound 3b. | S12 |
| Figure S15. GC-MS (NCI) of compound 3c. | S13 |
| Figure S16. GC-MS (NCI) of compound 3d. | S13 |
| Figure S17. GC-MS (NCI) of compound 3e. | S14 |
| Figure S18. GC-MS (NCI) of compound 3f. | S14 |

**Figure S1.** FT-IR spectrum of compound 3a.

**Figure S2.** FT-IR spectrum of compound 3b.

**Figure S3**. FT-IR spectrum of compound **3c**.

**Figure S4**. FT-IR spectrum of compound **3d**.

**Figure S5**. FT-IR spectrum of compound **3e**.

**Figure S6**. FT-IR spectrum of compound **3f**.

**Figure S7**. ^1^H-NMR spectrum of compound **3a** in DMSO-*d*_6_.

**Figure S8**. ^1^H-NMR spectrum of compound **3b** in DMSO-*d*_6_.

**Figure S9**. ^1^H-NMR spectrum of compound 3c in DMSO-*d*_6_.

**Figure S10**. ^1^H-NMR spectrum of compound 3d in DMSO-*d*_6_.

**Figure S11**.  ^1^H-NMR spectrum of compound 3e in DMSO-*d*_6_.

**Figure S12**. ^1^H-NMR spectrum of compound 3f in DMSO-*d*_6_.

**Figure S13**. GC-MS (NCI) of compound 3a.

**Figure S14**. GC-MS (NCI) of compound 3b.

**Figure S15.** GC-MS (NCI) of compound 3c.

**Figure S16.** GC-MS (NCI) of compound 3d.

**Figure S17.** GC-MS (NCI) of compound 3e.

**Figure S18.** GC-MS (NCI) of compound 3f.
